# Supplementary material for: Iterative improvement in the automatic modular design of robot swarms
Source: PeerJ Comput Sci. 2020 Dec 7;6:e322. doi: 10.7717/peerj-cs.322 (PMC7924708; doi:10.7717/peerj-cs.322)
Supplement: Supplemental Information 3 [file peerj-cs-06-322-s003.zip › argos3/doc/api/standalone/a00540_source.html]

ARGoS: plugins/robots/generic/simulator/quadrotor\_speed\_default\_actuator.cpp Source File


- Main Page
- Related Pages
- Namespaces
- Classes
- Files

- File List
- File Members

# plugins/robots/generic/simulator/quadrotor\_speed\_default\_actuator.cpp

Go to the documentation of this file.

```
00001 
00007 #include "quadrotor_speed_default_actuator.h"
00008 #include <argos3/core/utility/logging/argos_log.h>
00009 
00010 namespace argos {
00011 
00012    /****************************************/
00013    /****************************************/
00014 
00015    CQuadRotorSpeedDefaultActuator::CQuadRotorSpeedDefaultActuator() :
00016       m_pcQuadRotorEntity(NULL) {
00017    }
00018 
00019    /****************************************/
00020    /****************************************/
00021 
00022    void CQuadRotorSpeedDefaultActuator::SetRobot(CComposableEntity& c_entity) {
00023       try {
00024          /* Get the quadrotor component */
00025          m_pcQuadRotorEntity = &(c_entity.GetComponent<CQuadRotorEntity>("quadrotor"));
00026          /* Check whether the control methods is unset - only one is allowed */
00027          if(m_pcQuadRotorEntity->GetControlMethod() == CQuadRotorEntity::NO_CONTROL) {
00028             /* Get the robot body */
00029             m_pcEmbodiedEntity = &(c_entity.GetComponent<CEmbodiedEntity>("body"));
00030             /* Set the speed control method */
00031             m_pcQuadRotorEntity->SetControlMethod(CQuadRotorEntity::SPEED_CONTROL);
00032          }
00033          else {
00034             THROW_ARGOSEXCEPTION("Can't associate a quadrotor speed actuator to entity \"" << c_entity.GetId() << "\" because it conflicts with a previously associated quadrotor actuator.");
00035          }
00036       }
00037       catch(CARGoSException& ex) {
00038          THROW_ARGOSEXCEPTION_NESTED("Error setting quadrotor speed actuator to entity \"" << c_entity.GetId() << "\"", ex);
00039       }
00040    }
00041 
00042    /****************************************/
00043    /****************************************/
00044 
00045    void CQuadRotorSpeedDefaultActuator::Init(TConfigurationNode& t_tree) {
00046       try {
00047          CCI_QuadRotorSpeedActuator::Init(t_tree);
00048          Reset();
00049       }
00050       catch(CARGoSException& ex) {
00051          THROW_ARGOSEXCEPTION_NESTED("Initialization error in quadrotor speed actuator.", ex);
00052       }
00053    }
00054 
00055    /****************************************/
00056    /****************************************/
00057 
00058    void CQuadRotorSpeedDefaultActuator::SetLinearVelocity(const CVector3& c_velocity) {
00059       m_sDesiredSpeedData.Velocity = c_velocity;
00060    }
00061 
00062    /****************************************/
00063    /****************************************/
00064 
00065    void CQuadRotorSpeedDefaultActuator::SetRotationalSpeed(const CRadians& c_speed) {
00066       m_sDesiredSpeedData.RotSpeed = c_speed;      
00067    }
00068 
00069    /****************************************/
00070    /****************************************/
00071 
00072    void CQuadRotorSpeedDefaultActuator::Update() {
00073       m_pcQuadRotorEntity->SetSpeedControlData(m_sDesiredSpeedData);
00074    }
00075 
00076    /****************************************/
00077    /****************************************/
00078 
00079    void CQuadRotorSpeedDefaultActuator::Reset() {
00080       m_sDesiredSpeedData.Velocity = CVector3();
00081       m_sDesiredSpeedData.RotSpeed = CRadians::ZERO;
00082       Update();
00083    }
00084 
00085    /****************************************/
00086    /****************************************/
00087 
00088 }
00089 
00090 REGISTER_ACTUATOR(CQuadRotorSpeedDefaultActuator,
00091                   "quadrotor_speed", "default",
00092                   "Carlo Pinciroli [ilpincy@gmail.com]",
00093                   "1.0",
00094                   "The quadrotor speed actuator.",
00095                   "This actuator controls the speed of a quadrotor robot. For a\n"
00096                   "complete description of its usage, refer to the\n"
00097                   "ci_quadrotor_speed_actuator.h file.\n\n"
00098                   "REQUIRED XML CONFIGURATION\n\n"
00099                   "  <controllers>\n"
00100                   "    ...\n"
00101                   "    <my_controller ...>\n"
00102                   "      ...\n"
00103                   "      <actuators>\n"
00104                   "        ...\n"
00105                   "        <quadrotor_speed implementation=\"default\" />\n"
00106                   "        ...\n"
00107                   "      </actuators>\n"
00108                   "      ...\n"
00109                   "    </my_controller>\n"
00110                   "    ...\n"
00111                   "  </controllers>\n\n"
00112                   "OPTIONAL XML CONFIGURATION\n\n"
00113                   "None for the time being.\n\n"
00114                   ,
00115                   "Usable"
00116    );
```

---

Generated on 10 Jul 2018 for ARGoS by 
 1.6.1
